# Anti-Aging β-Klotho Gene-Activated Scaffold Promotes Rejuvenative Wound Healing Response in Human Adipose-Derived Stem Cells

**DOI:** 10.3390/ph14111168

**Published:** 2021-11-17

**Authors:** Ashang L. Laiva, Fergal J. O’Brien, Michael B. Keogh

**Affiliations:** 1 Tissue Engineering Research Group-Bahrain, Royal College of Surgeons in Ireland, Adliya, Manama P.O. Box 15503, Bahrain; lluwang@rcsi-mub.com; 2 Tissue Engineering Research Group, Department of Anatomy and Regenerative Medicine, Royal College of Surgeons in Ireland, 123 St. Stephen’s Green, D02 YN77 Dublin, Ireland; fjobrien@rcsi.ie; 3 Trinity Centre for Biomedical Engineering, Trinity Biomedical Sciences Institute, Trinity College Dublin, D02 PN40 Dublin, Ireland; 4 Advanced Materials and Bioengineering Research Centre, Royal College of Surgeons in Ireland and Trinity College Dublin, D02 YN77 Dublin, Ireland

**Keywords:** anti-aging, β-Klotho, gene-activated scaffold, adipose-derived stem cells, angiogenesis, matrix deposition, rejuvenative healing

## Abstract

Wound healing requires a tight orchestration of complex cellular events. Disruption in the cell-signaling events can severely impair healing. The application of biomaterial scaffolds has shown healing potential; however, the potential is insufficient for optimal wound maturation. This study explored the functional impact of a collagen-chondroitin sulfate scaffold functionalized with nanoparticles carrying an anti-aging gene β-Klotho on human adipose-derived stem cells (ADSCs) for rejuvenative healing applications. We studied the response in the ADSCs in three phases: (1) transcriptional activities of pluripotency factors (Oct-4, Nanog and Sox-2), proliferation marker (Ki-67), wound healing regulators (TGF-β3 and TGF-β1); (2) paracrine bioactivity of the secretome generated by the ADSCs; and (3) regeneration of basement membrane (fibronectin, laminin, and collagen IV proteins) and expression of scar-associated proteins (α-SMA and elastin proteins) towards maturation. Overall, we found that the β-Klotho gene-activated scaffold offers controlled activation of ADSCs’ regenerative abilities. On day 3, the ADSCs on the gene-activated scaffold showed enhanced (2.5-fold) activation of transcription factor Oct-4 that was regulated transiently. This response was accompanied by a 3.6-fold increase in the expression of the anti-fibrotic gene TGF-β3. Through paracrine signaling, the ADSCs-laden gene-activated scaffold also controlled human endothelial angiogenesis and pro-fibrotic response in dermal fibroblasts. Towards maturation, the ADSCs-laden gene-activated scaffold further showed an enhanced regeneration of the basement membrane through increases in laminin (2.1-fold) and collagen IV (8.8-fold) deposition. The ADSCs also expressed 2-fold lower amounts of the scar-associated α-SMA protein with improved qualitative elastin matrix deposition. Collectively, we determined that the β-Klotho gene-activated scaffold possesses tremendous potential for wound healing and could advance stem cell-based therapy for rejuvenative healing applications.

## 1. Introduction

Wound healing is a complex biological process that requires a tight orchestration of multiple cellular events [1]. However, in the aging population, cellular events are disrupted, leading to delayed healing [2]. The disruption in the healing mechanisms arises partly due to the reduced homing of the progenitor cells from the bone marrow and other prevailing metabolic diseases such as diabetes [3,4]. In wound management, the application of biomaterial scaffolds is becoming more common as a treatment. Biomaterial scaffolds protect the wound from infection, absorb wound exudates, and keep the wound moist to prevent tissue necrosis [5]. Therapeutics can also be loaded into biomaterial scaffolds and promote faster healing in hard-to-heal wounds [1]. However, biomaterial scaffolds alone may not effectively orchestrate the multiple signaling cascades occurring within the wound. Delivering stem cells is sometimes proposed as a solution for moderating the complex signaling events in the wound [6].

Stem cells secrete paracrine factors and can differentiate into cells of multiple tissue lineages [7]. Stem cells are typically delivered into the wound through intradermal injections [6], topical spraying [8], or as tissue-engineered grafts [9]. In recent years, the application of tissue-engineered grafts for chronic wound healing has gradually gained wider acceptance because of their ability to heal wounds rapidly [9,10,11]. Apligraf^®^ and Dermagraft^®^ are two of the widely used Food and Drug Administration (FDA)-approved bioengineered constructs for chronic wound treatment [12,13]. However, the generation of these grafts requires prolonged cell culture to produce high cell numbers [14]. When using stem cells, prolonged culture can increase cellular senescence and diminish the stemness of the stem cells [15,16]. Therefore, maintaining the stemness of the stem cells is central to achieving the optimal therapeutic response.

Therapeutic gene delivery to the stem cells using non-viral vectors is a potential strategy to enhance stem cells’ functionality [17]. Traditionally, the cells are transfected in 2 D cultures and later transplanted in vivo [18]. However, platforms such as the gene-activated scaffolds, consisting of biomaterial scaffolds functionalized with nanoparticles carrying the therapeutic transgene [19], that our group have been pioneering offer an alternative solution for transfecting the cells within a 3 D environment. This study focused on developing a gene-activated version of the 3 D collagen-chondroitin sulfate (coll-CS) scaffold that has tremendous clinical translation potential, as its composition is similar to that of the Integra’s Dermal Regeneration Template (DRT), a clinically approved scaffold for chronic wound healing [20]. We primarily use a cationic polymer called polyethyleneimine (PEI) to condense DNA plasmids encoding for growth factor genes such as the stromal-derived factor-1 alpha and nerve growth factor and assemble them into charged nanoparticles [21,22,23]. These charged nanoparticles are then soak-loaded into our coll-CS scaffold to generate the gene-activated scaffolds. Our previous studies have also shown that the PEI-based gene-activated scaffold can cause transient overexpression of the therapeutic transgene in a range of wound-healing cells and promote their regenerative abilities [21,24,25]. Traditionally, the gene-activated scaffold was developed to target host cells and promote local repair [26]. However, emerging evidence indicates that cell-laden gene-activated scaffolds bear superior potency to regenerate complex tissue structures than gene-activated scaffolds alone [27].

Cellular abundance is one of the crucial requirements for the generation of tissue-engineered grafts [28]. The adipose tissue is a rich source that can supply a large number of stem cells. The adipose tissue contains as many as 500-times the stem cells for the same mass of bone marrow, a traditional tissue source for stem cell extraction [29]. Moreover, the stem cells can be extracted from the adipose tissue using a minimally invasive liposuction process [29]. Our recent study found that the human adipose-derived stem cells (ADSCs) demonstrate excellent biocompatibility with the coll-CS scaffold. Using a pro-angiogenic gene-activated scaffold further improved the ADSCs’ regenerative responses [30]. However, the ADSCs tend to lose their stemness upon expansion [31]. Previously, we found that an anti-aging protein β-Klotho could significantly improve the proliferation of healthy and diabetic human ADSCs [32]. Most studies generally use the primary variant α-Klotho [33,34,35] and have shown that the protein can increase the lifespan of ADSCs by activating the telomerase transcriptional activity [33]. α-Klotho overexpressing MSCs have also been found to exert strong anti-fibrotic effects in kidney injury through the inhibition of Wnt/β-catenin signaling [36]. However, the role of β-Klotho in stem cells remains relatively unexplored.

Besides the difference in the research focus between the α- and β-Klotho, the anti-aging Klotho proteins have not yet been fully incorporated into the tissue engineering strategies for wound healing. Therefore, in this study, we sought to study the functional impact of a β-Klotho gene-activated scaffold on human ADSCs for wound healing applications. We first investigated the transcriptional activities of the classical pluripotency factors (Oct-4, Nanog and Sox-2), proliferation marker (Ki-67) and wound healing regulators (TGF-β3 and TGF-β1) in the ADSCs. We then assessed the bioactivity of the conditioned media generated by the ADSCs-laden gene-activated scaffold to evaluate its paracrine potential. Ultimately, we examined the regeneration of basement membrane (fibronectin, laminin, and collagen IV) and scar-associated proteins (α-SMA and elastin) in the ADSCs-laden gene-activated scaffold to evaluate controlled tissue maturation.

## 2. Results

### 2.1. β-Klotho Gene-Activated Scaffold Transiently Enhances Human ADSCs’ Stemness and Pro-Reparative Genes

**Figure 1:**
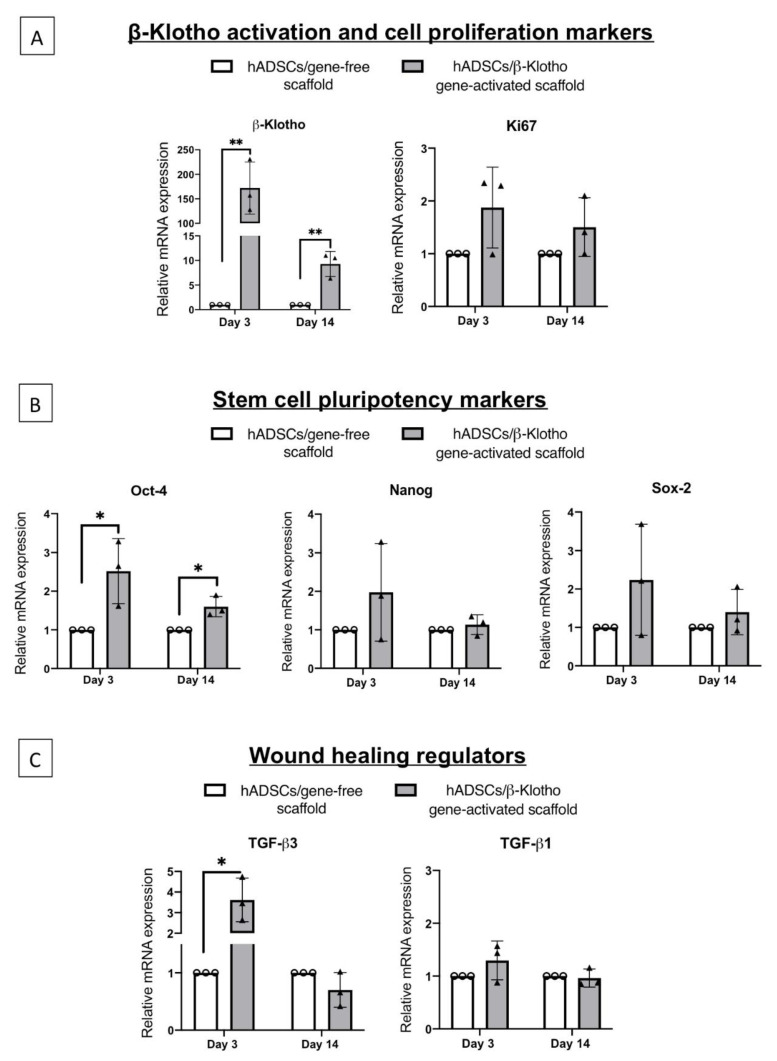
Transcriptional activation of functional genes in the human ADSCs by β-Klotho gene-activated scaffold on day 3 (early) and day 14 (aged). (**A**) The ADSCs on the gene-activated scaffold showed a transient, high-level overexpression of the therapeutic transgene β-Klotho until 14 days. Ki-67 marker indicates that the ADSCs proliferated well within the gene-activated scaffold. (**B**) β-Klotho overexpression significantly induced the activation of pluripotency factor Oct-4 in the ADSCs while maintaining basal levels of Nanog and Sox-2. (**C**) Increased activation of the stemness in the ADSCs was further associated with a robust early activation of the anti-fibrotic gene TGF-β3, while maintaining basal levels of the pro-fibrotic TGF-β1. ** and * indicates *p* < 0.01 and *p* < 0.05 respectively. Data represents mean ± standard deviation (*n* = 3).

Gene expression analysis first showed that the ADSCs on the gene-activated scaffold overexpressed the β-Klotho gene over 172-fold (*p* < 0.01) than the gene-free scaffold group on day 3 (Figure 1A). This finding confirmed the efficient interaction of the ADSCs with the gene-activated scaffold. Analysis of the proliferation marker Ki-67 further confirmed that the ADSCs on the gene-activated scaffold also maintained a robust proliferative capacity over 14 days (Figure 1A).

Having confirmed the overexpression of the anti-aging β-Klotho gene, we then assessed stem cells’ rejuvenation through the activation of “pluripotency” factors in the ADSCs. Overall, we noted a transient regulation of all the three transcription factors over the 14 days (Figure 1B). However, the ADSCs only showed a significant increase in the expression of the Oct-4 gene that sustained until day 14. Specifically, the ADSCs showed a 2.5-fold (*p* < 0.05) increase in the expression of the Oct-4 gene on day 3 before subsiding to 1.6-fold (*p* < 0.05) on day 14 relative to the gene-free scaffold group.

The next set of genes we investigated were the transforming growth factors beta 1 and 3, which are crucial for regulating scarless wound healing (Figure 1C). The levels of pro-fibrotic TGF-β1 gene in the β-Klotho overexpressing ADSCs were found to be in par with that of the gene-free scaffold group over the 14 days. However, the levels of anti-fibrotic TGF-β3 were significantly (3.6-fold, *p* < 0.05) elevated during the early days (day 3) and, as anticipated, subsided by about 0.4-fold by day 14, showing a controlled expression of the regulatory gene.

### 2.2. β-Klotho Gene-Activated Scaffold Enhances the Paracrine Potency of Human ADSCs

#### 2.2.1. Pro-Angiogenic Bioactivity

**Figure 2:**
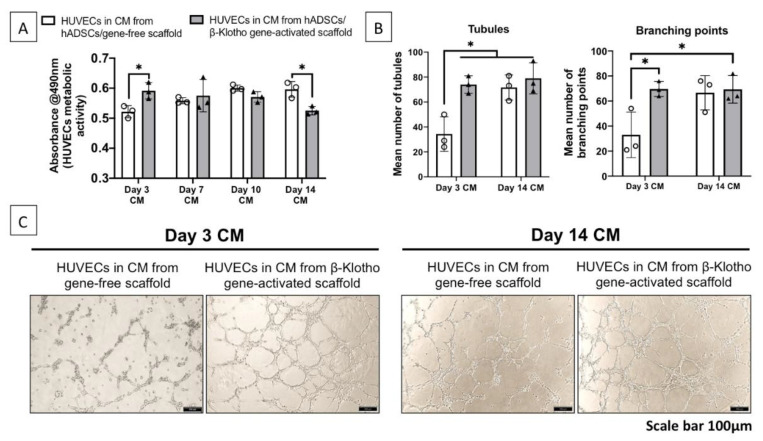
Pro-angiogenic paracrine bioactivity of the ADSCs-laden gene-activated scaffold CM. (**A**) CM produced by the ADSCs-laden gene-activated scaffold over the 14 days demonstrated an ability to temporally control HUVECs’ metabolic activity. (**B**) Day 3 CM from the gene-activated scaffold group significantly enhanced endothelial tubule formation and its branching compared to that of the gene-free scaffold group. (**C**) Tubule formation and branching of endothelial cells 6 h post-exposure to CM from the ADSCs seeded gene-activated and gene-free scaffold. * indicates *p* < 0.05. Data represents mean ± standard deviation (*n* = 3).

The bioactivity test of the CM generated by ADSCs-laden gene-activated scaffold first showed that it could temporally control the metabolic activity in HUVECs. Day 3 CM significantly enhanced the metabolic activity in HUVECs, while the use of aged CM from day 14 significantly reduced the HUVEC’s metabolic activity (Figure 2A). Having observed this, we then tested the pro-angiogenic impact of the CM from day 3 and 14 on the HUVECs seeded on Matrigel. As anticipated, day 3 CM from the gene-activated scaffold group significantly (*p* < 0.05) enhanced HUVEC tubule formation capacity and their branching (Figure 2B). However, no further rise in the angiogenic activity occurred with day 14 CM compared to that of the day 3 CM. Also, the day 14 CM from both the groups exhibited similar pro-angiogenic efficiency (Figure 2C). Collectively, our finding indicates that the gene-activated scaffold transiently expedites ADSCs’ pro-angiogenic response.

#### 2.2.2. Dermal Fibroblast Healing and Maturation

**Figure 3:**
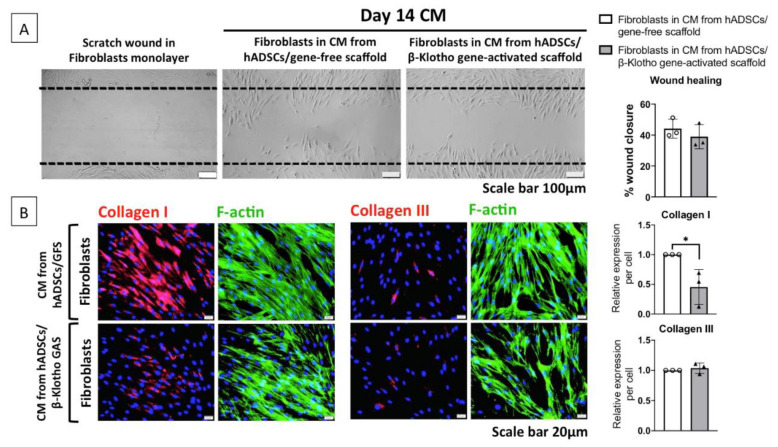
Paracrine influence on dermal healing and maturation. (**A**) Aged day 14 CM from both the groups exerted similar levels of wound closure activity in human adult dermal fibroblasts by approximately 40% in 12 h. (**B**) CM from the gene-activated scaffold significantly reduced the expression of pro-fibrotic collagen I in the fibroblasts. * indicates *p* < 0.05. Data represents mean ± standard deviation (*n* = 3). In Figure 3B, GAS and GFS stand for gene-activated scaffold and gene-free scaffold respectively.

One of the findings from the angiogenesis study is that as the ADSCs-laden gene-activated scaffold matures, it no longer promotes angiogenesis. Furthermore, on day 14, their angiogenic potency was similar to that of the gene-free scaffold group (Figure 2B,C). Therefore, we investigated the influence of aged day 14 CM on dermal fibroblast wound closure. Similar to the angiogenesis study result, both groups demonstrated similar levels of fibroblast wound closure with day 14 CM. Specifically, both the groups healed the wound by approximately 40% in 12 h (Figure 3A). This finding revealed that during maturation, the gene-activated scaffold group supports but does not promote dermal fibroblasts’ wound closure.

Nevertheless, as the fibroblasts were stimulated with aged CM, we studied if the CM influences the expression of matrix proteins involved in wound maturation. As anticipated, dermal fibroblasts stimulated with CM from the gene-free scaffold group abundantly expressed the pro-fibrotic collagen I protein (Figure 3B). On the contrary, fibroblasts stimulated with CM from the gene-activated scaffold group demonstrated 50% lower expression (*p* < 0.05) of the collagen I protein. Meanwhile, both the groups lacked the expression of anti-fibrotic collagen III, and no differences were observed between the two groups. Taken together, it implies that the gene-activated scaffold enhances ADSCs’ wound modulatory response during maturation.

### 2.3. β-Klotho Gene-Activated Scaffold Enhances Basement Membrane Regeneration with Improved Anti-Fibrotic Response in Human ADSCs

**Figure 4:**
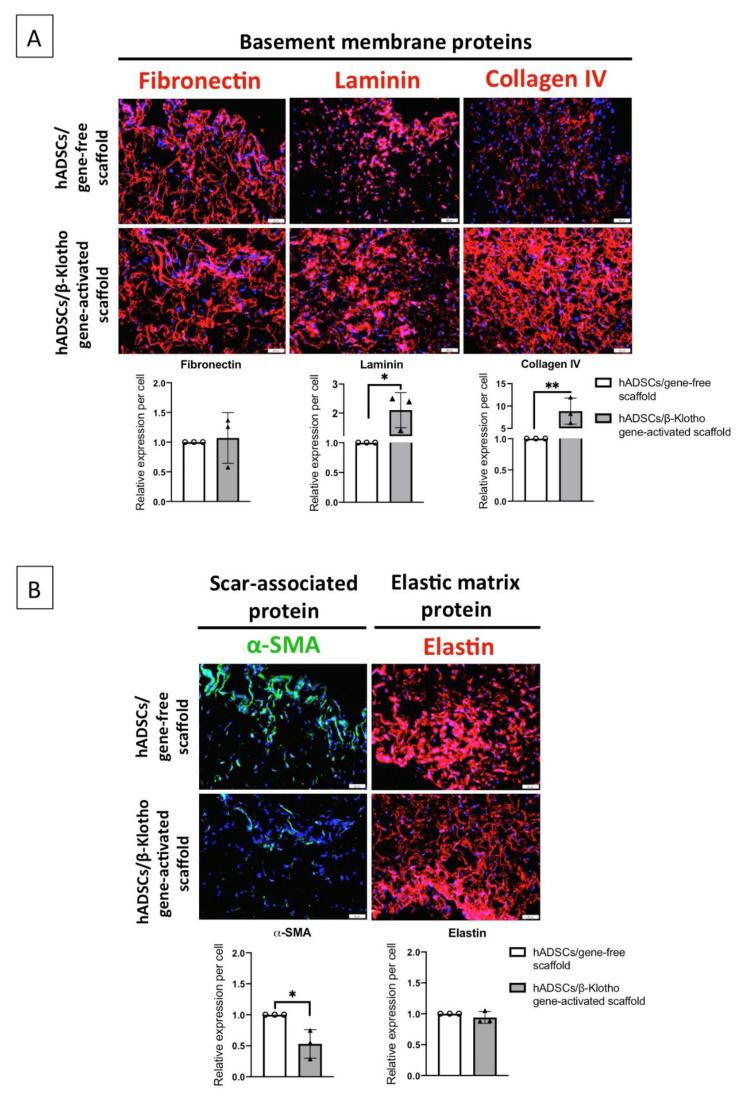
Deposition of pro-wound healing matrix proteins by the ADSCs in the gene-activated scaffold on day 14. (**A**) The ADSCs in the gene-activated scaffold demonstrated significant regeneration of the basement membrane compared to the ADSCs in the gene-free scaffold. The ADSCs predominantly deposited a relatively mature network of collagen IV proteins, followed by laminin and fibronectin. (**B**) The ADSCs in the gene-activated scaffold also expressed 2-fold lower of the α-SMA protein compared to the ADSCs in the gene-free scaffold. In conjunction with basement membrane regeneration, the ADSCs in the gene-activated scaffold deposited qualitatively better elastin matrix. All the images were captured through 20× objective using an IX73 Olympus microscope. ** and * indicates *p* < 0.01 and *p* < 0.05 respectively. Scale bar 50μm. Data represents mean ± standard deviation (*n* = 3). α-SMA and fibronectin was double-immunostained.

Having observed the gene-activated scaffold’s controlled stimulation of ADSCs’ functionality, we ultimately investigated the ADSCs’ response towards the regeneration of dermal matrices and proteins. The gene-activated scaffold robustly enhanced the regeneration of ADSCs’ basement membrane. Specifically, the gene-activated scaffold significantly promoted the deposition of basement membrane components laminin and collagen IV (Figure 4A). After the semi-quantitative analysis, we also noted that the matrix deposition followed a trend relative to the gene-free scaffold group. The trend as observed was fibronectin (1.0-fold) < laminin (2.1-fold, *p* < 0.05) < collagen IV (8.8-fold, *p* < 0.01).

Once the ability to regenerate the basement membrane was determined, we then assessed the expression of proteins crucial for superior qualitative healing, such as reduced scarring. The ADSCs on the gene-activated scaffold demonstrated a significantly (*p* < 0.05) reduced expression of the scar-associated contractile protein α-SMA. The α-SMA expression in the ADSCs was lower by 50% relative to the gene-free scaffold group (Figure 4B). Lastly, having observed the reduced α-SMA expression, we assessed the expression of the elastic matrix protein elastin. The ADSCs on the gene-activated scaffold demonstrated a relatively superior qualitative elastin expression through the deposition of mature fibrous network of elastin compared to the ADSCs on the gene-free scaffold (Figure 4B).

## 3. Discussion

**Figure 5:**
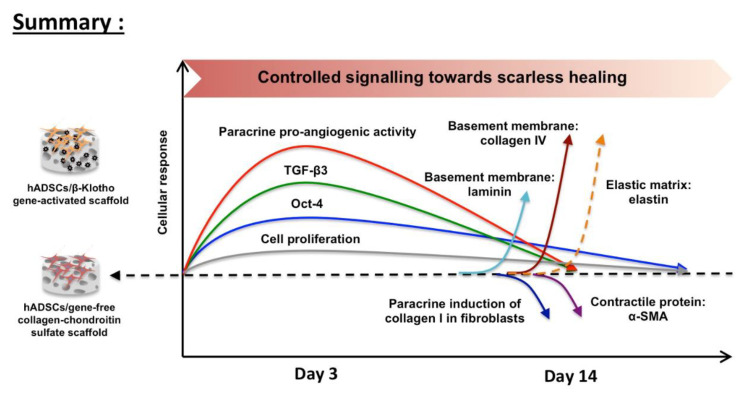
A schematic of the functional impact of β-Klotho gene-activated scaffold on human ADSCs for wound healing applications. The key findings of this study are the (1) transient enhancement of stem cell pluripotency and anti-fibrotic response, (2) improved paracrine control towards angiogenesis, and pro-fibrotic collagen remodeling in dermal fibroblasts, and ultimately, (3) increased maturation of the basement membrane with control over scar-associated proteins’ expression. Dotted lines for elastin matrix imply improved qualitative deposition.

This study aimed to investigate the functional impact of an anti-aging β-Klotho gene-activated scaffold on human ADSCs for enhanced wound healing applications. Overall, we found that the gene-activated scaffold offers controlled activation of ADSCs’ regenerative abilities as depicted in Figure 5. Specifically, the gene-activated scaffold transiently enhanced ADSCs’ stemness through the activation of transcription factor Oct-4. The gene-activated scaffold also promoted early activation of the anti-fibrotic gene TGF- β3, a crucial factor for controlled healing with reduced scarring. Furthermore, the activated ADSCs temporally controlled endothelial angiogenesis and supported dermal fibroblasts’ healing through paracrine signaling. Towards maturation, the ADSCs also controlled the activation of pro-fibrotic response in the dermal fibroblasts. Meanwhile, the ADSCs on the gene-activated scaffold effectively regenerated the dermal basement membrane by enhancing laminin and collagen IV deposition. This response was further associated with reduced scar-associated α-SMA protein expression and improved qualitative elastin matrix deposition. Our study collectively determined that the β-Klotho gene-activated scaffold possesses tremendous potential for wound healing and could advance stem cell-based therapy for rejuvenative healing applications.

In wound healing strategies, plasmid-based gene therapy is applied to transiently enhance cellular responses until wound repair is complete [37]. Our gene-activated scaffold also shows that the therapeutic β-Klotho gene is transiently regulated over 14 days. The significantly enhanced (170-fold) expression of the β-Klotho mRNA at day 3 could be attributed to the immediate-early CMV promoter that is known to enhance transcriptional activation of the encoded transgene [38]. However, in the MSCs, the CMV could undergo DNA methylation or histone deacetylation that can reduce the transgene expression [39]. Moreover, the plasmids generally are non-replicating episomes, limiting the transfer of transgene to dividing cells, causing a decline in the transgene expression over time [40]. The presence of heterochromatic markers originating from the bacterial sequences of the plasmid could further repress transgene expression [41], overall contributing to a transient gene expression.

One of the avenues pursued for the treatment of hard-to-heal wounds is the application of cell-seeded bioactive scaffolds. One reason is their ability to promote rapid wound repair. Metabolically active cells in the graft secrete paracrine factors and undergo differentiation, orchestrating the complex signaling events in the wound environment [42]. Stem cells, in particular, have a unique ability to sense external stimuli and modulate their response to restore homeostasis [43]. However, stem cells lose their stemness when they are expanded in vitro to generate large cell numbers for the graft. Our study shows that the stemness can be transiently enhanced using a gene-activated scaffold carrying an anti-aging gene β-Klotho. Specifically, we noted that the anti-aging gene-activated scaffold enhanced the expression of the stemness gene Oct-4 in the ADSCs. The Oct-4 is one of the key transcriptional factors in embryonic stem cells and is essential for controlled embryonic development [44]. In the ADSCs, the activation of the Oct-4 gene can promote their proliferation and differentiation potential [45]. Thus, the increased expression of Oct-4 may facilitate rapid differentiation of the ADSCs on the gene-activated scaffold into host cells and aid in the healing process.

Having observed the activation of Oct-4 gene in the ADSCs, we then assessed the angiogenic potency of the ADSCs. Angiogenesis is crucial for efficient integration of the graft with the host tissue [46]. Cells in the graft trigger angiogenesis through the secretion of paracrine factors. Angiogenesis is also a crucial event for granulation tissue development and its activity diminishes as the granulation tissue mature [47]. This programmed limitation of the angiogenic activity at the healing’s later stages is essential for controlling scarring and hypergranulation that can impede re-epithelialization [48]. Our finding also shows that the ADSCs on the gene-activated scaffold can enhance endothelial sprouting and control their growth through paracrine signaling. Additionally, the significant increases in the endothelial cells’ metabolic activity and sprouting in response to day 3 CM demonstrate the potential of the ADSCs-laden gene-activated scaffold to integrate with the host tissue rapidly. Moreover, the use of stem cells CM is a commonly adopted cell-free approach to enhance quality wound healing [49]. As a limitation, we acknowledge that adult dermal endothelial cells would serve a better cell model for studying angiogenesis; however to maintain consistency with our previous studies and the HUVECs being widely accepted as the “gold-standard” cell candidate [50], we adopted the HUVECs angiogenesis assay.

An increase in fibroblasts’ remodeling activity is a general feature of granulation tissue maturation [51]. During this stage, collagen III matrix is gradually replaced by stronger collagen I fibers [51]. Collagen fibers then promote wound contraction [52], and wound contraction is indispensable for complete wound closure [53]. However, an aggressive sustained increase in collagen I deposition can increase scar formation [54]. Controlling scarring is crucial as it can inhibit the development of other skin appendages such as hair follicles, sebaceous glands, and sweat glands, for example, in burn wounds [54]. Our finding demonstrates that the aged (day 14) CM from the gene-activated scaffold group could control the expression of pro-fibrotic collagen I in the adult dermal fibroblasts. This controlled effect towards the fibroblasts occurred without further influence towards the fibroblast’s migration or their anti-fibrotic collagen III expression relative to the gene-free scaffold group. Therefore, we suggest that the ADSCs-laden gene-activated scaffold may offer better control in limiting scarring in vivo through controlled collagen I expression. Li et al., have also shown that the ADSCs CM can significantly reduce collagen I expression in fibroblasts derived from hypertrophic scars [55]. Another study by Wang et al., demonstrated similar reduction in collagen I expression in keloid-derived fibroblasts [56], collectively demonstrating the anti-fibrotic potential of the ADSCs’ CM.

One of the significant findings that we noted with the application of the β-Klotho gene-activated scaffold is the enhanced regeneration of basement membrane in the ADSCs. The regeneration of the basement membrane is essential for blood vessel maturation and complete re-epithelialization [57,58]. Importantly, the trend in which the proteins laminin (2.1-fold) and collagen IV (8.8-fold) are upregulated further indicates increased maturation of the basement membrane in the gene-activated scaffold group [59]. Besides acting as an anti-aging factor [60], the beta klotho functions to enhance sensitiveness to fibroblast growth factor (FGF) by FGF receptors [61]. Additionally, increase in FGF signaling has been found to promote basement membrane maturation through the deposition of laminin and collagen IV [62]. Therefore, considering beta klotho’s role as a co-receptor of FGF, the increased basement maturation in the gene-activated scaffold group is potentially mediated by increase in ADSCs’ FGF signaling.

The basement membrane component remarkably upregulated in the gene-activated scaffold group is the collagen IV protein. The collagen IV protein represents 50% of all basement membrane [63] and provides structural stability to the basement membrane [59]. However, aging significantly reduces collagen IV expression in the dermal-epidermal junction [58,64] and the lack of collagen IV can promote scar pathogenesis [65]. Thus, our results suggest that the increased basement membrane regenerative potency of the ADSCs-laden gene-activated scaffold can significantly aid in enhanced healing in aged patients. Other studies have also shown that the adipose tissue-derived ECM possess tremendous therapeutic potential for skin repair [66]. Furthermore, tissue-engineered grafts rich in laminin and collagen IV is also of interest for respiratory epithelium repair [67].

In addition to the pro-fibrotic control towards the fibroblasts, our study further shows that the ADSCs in the gene-activated scaffold also express a 2-fold lower expression of the scar-associated protein α-SMA [68]. Although we did not evaluate its impact in vivo, the reduction in local α-SMA expression is crucial for improved qualitative healing [69]. Using fibrotic agents such as bleomycin [70] could be one way to evaluate better the anti-fibrotic properties of the ADSCs/gene-activated scaffold construct in vitro; however, our focus was to establish the provisional therapeutic potential of the construct.

Another indicator that the ADSCs-laden gene-activated scaffold may improve qualitative healing is the deposition of fibrous elastin matrix instead of patchy extracellular secretions in the gene-free scaffold group. Elastin deposition is one of the main activities that drive fetal scarless wound healing [71]. However, adult skins lack the deposition of elastin [71]. As such, tropoelastin, a precursor of elastin, is often incorporated into biomaterial scaffolds to control scarring in wound healing [72]. Taken together, our findings imply that the ADSCs/β-Klotho gene-activated scaffold construct may confer a strong anti-fibrotic response in vivo.

## 4. Materials and Methods

### 4.1. Preparation of Gene-Activated Scaffold

The gene-activated scaffold was developed using a 2-step process. Firstly, solid porous collagen-chondroitin sulfate scaffolds were fabricated by freeze-drying slurry of bovine tendon type 1 collagen and chondroitin-6-sulfate derived from shark cartilage (Sigma, UK). An optimized freeze-drying process designed to create uniform pores were used to produce the scaffolds [73]. Based on our published protocol [74], the freeze-dried scaffolds were then dehydrothermally (DHT) treated at 105 °C under vacuum for sterilization and mechanical improvement. The sterilized scaffolds were then chemically cross-linked with 14 mM N-(3-Dimethylaminopropyl)-N’-ethylcarbodiimide hydrochloride and 5.5 mM N-Hydroxysuccinimide (2.5 molar ratio of EDC/NHS) (Sigma, UK) solution to further improve the mechanical stability [75]. The cross-linked scaffolds were then washed with PBS (Gibco, Paisley, UK) to remove residual chemicals. Once the scaffolds were ready, polyplexes at an N/P10 ratio (nitrogen to phosphate ratio) were prepared by mixing a predetermined volume of branched polyethyleneimine (PEI) solution with plasmid DNA (pDNA) encoding for the human beta-Klotho gene (β-Klotho), obtained from SinoBiological, Beijing, China. The N/P10 ratio was chosen based on our previous studies that found that polyplexes formulated at an N/P 10 ratio could effectively form small, stable cationic nanoparticles with plasmids as large as GLuc (5.76 kb) [21,23]. Prior to use, the plasmids were diluted in endotoxin free water to obtain a working concentration of 0.5 μg/μL. The plasmid contains human enhanced immediate-early cytomegalovirus (CMV3) promoter to promote high-level stable and transient expression of the encoded gene in mammalian cells.

The plasmid/PEI mix was allowed to settle for 30 min to self-assemble into polyplex nanoparticles. The nanoparticles were then soak-loaded onto the scaffolds by pipetting equal volumes of the polyplex solution per side of the scaffold. A total of 2 μg pDNA was used per scaffold to develop the gene-activated scaffold.

### 4.2. Cell Seeding on β-Klotho Gene-Activated Scaffold

Human ADSCs (iXCells Biotechnologies, San Diego, CA, USA) were expanded to passage 4 in the ADSCs growth medium (Cat no. MD0003) supplied by the company. A total of 5 × 10^5^ ADSCs (2.5 × 10^5^ per side) were then seeded per gene-free scaffold (control, *n* = 3) or gene-activated scaffold (test, *n* = 3). After letting the cells settle for about 20 min, 2 mL of transfection media OptiMEM (Gibco, UK) was added, and the cellularized scaffolds were incubated at 37 °C for 24 h. After the 24 h incubation, the cellularized gene-free or gene-activated scaffolds were transferred into new 12-well plates, and fed with 2 mL of ADSCs growth medium. Media change was then performed every 3–4 days until day 14 by collecting 1 mL of the conditioned media (CM) and replacing it with equal volume of fresh media. All CM were stored at −80 °C until analysis.

### 4.3. qRT-PCR Analyses to Determine β-Klotho Gene Overexpression and Activation of Functional Genes

In order to determine transient regulation of the target genes, cells were harvested at days 3 (early) and 14 (late) post-seeding on the gene-free or gene-activated scaffolds. The cells were first lysed using the Qiazol lysis reagent (Qiagen, Germantown, MD, USA). Chloroform was then added to separate the cell lysate into protein, DNA and RNA phases. Using the RNeasy Kit (Qiagen, Manchester, UK), the RNA was extracted, and their quality and quantity were determined using a Multiskan Go plate reader (Thermo Scientific, UK) with the absorbance set at 260 nm. Genomic DNA was then removed by mixing the RNA with a genomic DNA wipeout buffer (Qiagen, Manchester, UK) and heating to 42 °C for 2 min. Subsequently, reverse transcription was performed to prepare the cDNA. Duplicates of cDNA per replicate (*n* = 3) were loaded into the qRT-PCR plates and then the assay was run using the primers listed in Table 1. Fold change in mRNA expression relative to the cells on gene-free scaffold was calculated using the 2^−∆∆CT^ method from averages of three replicates per group. Human GAPDH (Hs_GAPDH_1_SG, Cat. No. QT00079247) was used as the housekeeping gene.

### 4.4. Bioactivity Analyses of Secreted Factors from the ADSCs on β-Klotho Gene-Activated Scaffold

#### 4.4.1. Pro-Angiogenic Bioactivity Analyses

Next, we studied the paracrine potency of the ADSCs on β-Klotho gene-activated scaffold. As angiogenesis is crucial for graft integration, we first studied the angiogenic impact of CM on human umbilical vein endothelial cells (HUVECs). The assessment was conducted in two stages. Firstly, a temporal effect of the CM on the HUVECs was determined by assaying the metabolic activity in the HUVECs incubated with the CM collected from all the time points—day 3, 7, 10, and 14. Briefly, 1 × 10^4^ HUVECs/well in a 96-well plate was incubated in the CM for 24 h and the response was assayed using the standard MTS assay. Subsequently, CM from days 3 and 14 were used for Matrigel assay. For the Matrigel assay, the HUVECs were seeded at a density of 3 × 10^4^ cells/well of a 48-well plate pre-coated with 120 μL of Matrigel for 30 min at 37 °C. At 6 h post-exposure to CM, images of the morphological changes in the endothelial cells were captured using an inverted microscope (IX73, Olympus, Japan) and the mean number of tubules and branching points were counted using the ImageJ software (ImageJ, National Institutes of Health, Bethesda, MD, USA).

#### 4.4.2. Dermal Fibroblasts Healing and Maturation Analyses

After the angiogenesis assay, we investigated the CM’s potency for dermal healing by employing a scratch assay of human adult dermal fibroblasts (iXcells, Cat no. 10 HU-014). For this study, we specifically chose the aged CM from day 14 to study its influence on fibroblasts’ wound closure during maturation. A total of 1 × 10^4^ cells/well in a 96-well plate were seeded and incubated overnight in the fibroblast growth medium. The next day, a wound was created on the monolayer by horizontally scratching a 200 uL pipette tip across the well. The monolayer was then rinsed with PBS to remove any debris and fed with the CM. Wound closure was recorded between 12–16 h after the scratch. Immunostaining was used to study the expression of pro-fibrotic collagen I (1:100, Novusbio, UK) and anti-fibrotic collagen III (1:100, Novusbio, UK) in the fibroblasts. The fibroblasts were counterstained with rhodamine (1:800, Abcam, UK) for F-actin imaging. Immunostaining was performed as described in Section 4.5, with the exception of tissue processing and deparafinization. Both HUVECs and the fibroblasts were used at passage 4.

### 4.5. Immunofluorescence Imaging of Extracellular Matrix Proteins

After 14 days of culture, the cellularized gene-free or gene-activated scaffolds were harvested for detection of matrix deposition using immunofluorescence. The processing of the samples was performed as described previously. Briefly, the scaffolds were first washed with PBS and fixed in 10% neutral buffered formalin for 20 min. The fixed samples were then processed using the standard protocol for paraffinization. The blocks were then cut into 7-μm thick slices and collected on charged slides. The sections were then deparaffinized using xylene followed by rehydration of the section with decreasing gradients of ethanol. Subsequently, the cells were permeabilized with 0.2% Tween^®^20 (Sigma-Aldrich, France) solution in PBS for 30 min (10 min wash × 3) and blocked using 10% NGS (Normal Goat Serum, Invitrogen, Rockford, IL, USA)/5% BSA/0.3 M Glycine (prepared in permeabilizing solution) for 1 h. After blocking, the slides were rinsed in PBS and then incubated at 4 °C overnight with the antibodies to target matrix proteins listed in Table 2.

The next day, the slides were rinsed in PBS thrice for 2–3 min each to remove any unbound primary antibodies. Subsequently, the slides were incubated in either Alexa 488-conjugated goat anti-mouse IgG (A32723, Invitrogen, UK) or Alexa 594-conjugated goat anti-rabbit IgG (A11012, Invitrogen, UK) at 1:800 dilution at room temperature for 1 h in the dark. The rinsing step was performed as before and counterstained for nuclei using the mounting medium with DAPI (ab104139, Abcam, UK). The slides were then imaged using fluorescence microscope (Olympus BX43, Japan) at 20× objective. Samples incubated with only secondary antibodies were used as controls.

#### Image Quantification

ImageJ software (ImageJ, NIH, MD, USA) was used to semi-quantitatively determine the amount of expressed proteins. For each marker, a constant threshold value was first determined through preliminary imaging of various sections. Using the set threshold value, integrated density (stained area × mean gray value) of the images was determined and then normalized to the number of cells (DAPI counting) to give a final mean fluorescence density per cell. An average was quantified from 8–10 random images per replicate, with a minimum of three replicates per group. The averages obtained from the three replicates/group were then used for measuring relative expression between the groups.

### 4.6. Statistical Analysis

All results are expressed as mean  ±  standard deviation. Unpaired, two-tailed t-test was generally used to calculate the statistical significance between groups, where *p* <  0.05 was considered to be significant.

## 5. Conclusions

This study showed that the anti-aging β-Klotho gene-activated scaffold offers controlled activation of the regenerative capacity of ADSCs. The key findings of this study are the (1) transient enhancement of stem cell pluripotency and anti-fibrotic response, (2) improved paracrine control towards angiogenesis, and pro-fibrotic collagen remodeling in dermal fibroblasts, and ultimately, (3) increased maturation of the basement membrane with control over scar-associated proteins’ expression. Conclusively, these results suggest that the ADSCs-laden β-Klotho gene-activated scaffold may possess great potential a treating a wide range of dermal wounds.

## Figures and Tables

**Table 1:** List of functional genes associated with ADSCs’ growth and development.

Function	Primer (Catalog No.)	Encoded Gene
Activation and proliferation	Hs_KLB_4_SG (QT02454977)	Beta Klotho (β-Klotho)
	Hs_MKI67_1_SG (QT00014203)	Marker of proliferation (Ki-67)
Stemness or pluripotency promoters	Hs_POU5 F1_1_SG (QT00210840)	Octamer-binding transcription factor 4 (Oct-4)
	Hs_NANOG_1_SG (QT01025850)	Homeobox protein (Nanog)
	Hs_SOX2_1_SG (QT00237601)	Sex determining region Y-box 2 (Sox-2)
Wound healing regulators	Hs_TGFB3_1_SG (QT00001302)	Transforming growth factor beta 3 (TGF-β3)
	Hs_TGFB1_1_SG (QT00000728)	Transforming growth factor beta 1 (TGF-β1)

**Table 2:** List of primary antibodies to extracellular matrix proteins involved in wound healing.

Functional Roles	Primary Antibodies (Catalog No.)	Dilutions in 1% BSA Solution
Basement membrane proteins	Fibronectin (ab2413, Abcam, UK)	1:200
	Laminin (ab11575, Abcam, UK)	1:200
	Collagen IV (ab6586, Abcam, UK)	1:200
Scar-associated contractile protein	Alpha-smooth muscle actin (ab7817, Abcam, UK)	1:100
Elastic matrix protein	Elastin (ab21607, Abcam, UK)	1:200

## Data Availability

The data presented in this study are available on request from the corresponding author. The data are not publicly available due to privacy or ethical restrictions.
